# Curcumin to inhibit binding of spike glycoprotein to ACE2 receptors: computational modelling, simulations, and ADMET studies to explore curcuminoids against novel SARS-CoV-2 targets

**DOI:** 10.1039/d0ra03167d

**Published:** 2020-08-25

**Authors:** Dhivya Shanmugarajan, Prabitha P., B. R. Prashantha Kumar, B. Suresh

**Affiliations:** Department of Pharmaceutical Chemistry, JSS College of Pharmacy, JSS Academy of Higher Education & Research Mysuru 570 015 India brprashanthkumar@jssuni.edu.in +91-821-2548359 +91-821-2548353; JSS Academy of Higher Education & Research Mysuru 570 015 India

## Abstract

The recent emergence of the novel coronavirus (SARS-CoV-2) has raised global concern as it is declared a pandemic by the WHO. However, to date, there is no current regimen to mitigate the molecular pathogenesis of SARS-CoV-2 virus. Curcuminoids, bioactive ingredients present in *Curcuma longa* (turmeric), are known to exhibit diverse pharmacological properties. To the best of our understanding to date, SARS-CoV-2 uses angiotensin-converting enzyme 2 (ACE2) for the host cellular entry. This is mediated *via* proteins of SARS-CoV-2, especially the spike glycoprotein receptor binding domain. Accordingly, our primary objective is to thwart virus replication and binding to the host system, leading us to probe curcuminoids efficiency towards key surface drug target proteins using the computational biology paradigm approach. Specifically, fourteen natural curcuminoids were studied for their possibility of inhibiting SARS-CoV-2. We studied their *in silico* properties towards SARS-CoV-2 target proteins by homology modelling, ADME, drug-likeness, toxicity predictions, docking molecular dynamics simulations and MM-PBSA free energy estimation. Among the curcuminoids docked to the receptor binding domain of SARS-CoV-2 spike glycoprotein, the keto and enol forms of curcumin form strong hydrogen bond interactions with ACE2 binding residues Q493, T501, Y505, Y489 and Q498. Molecular dynamics simulations, free energy binding and interaction energy validated the interaction and stability of the docked keto and enol forms of curcumin.

## Abstract 2

The significant role of curcumin against SARS-CoV-2 drug targets to thwart virus replication and binding into the host system using the computational biology paradigm approach.

## Graphical abstract

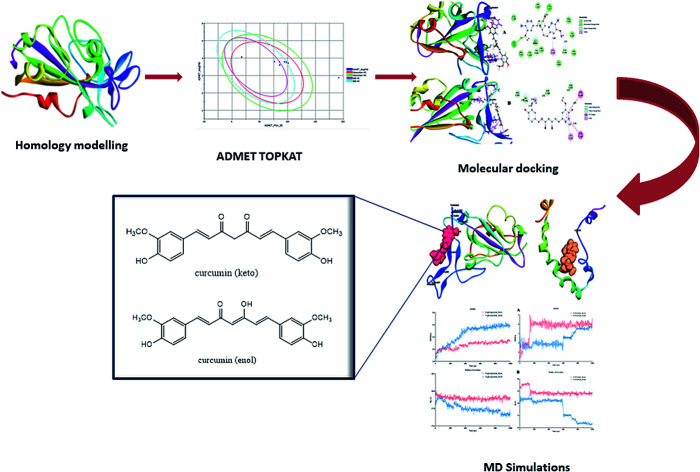

## 1. Introduction

**Fig. 1:**
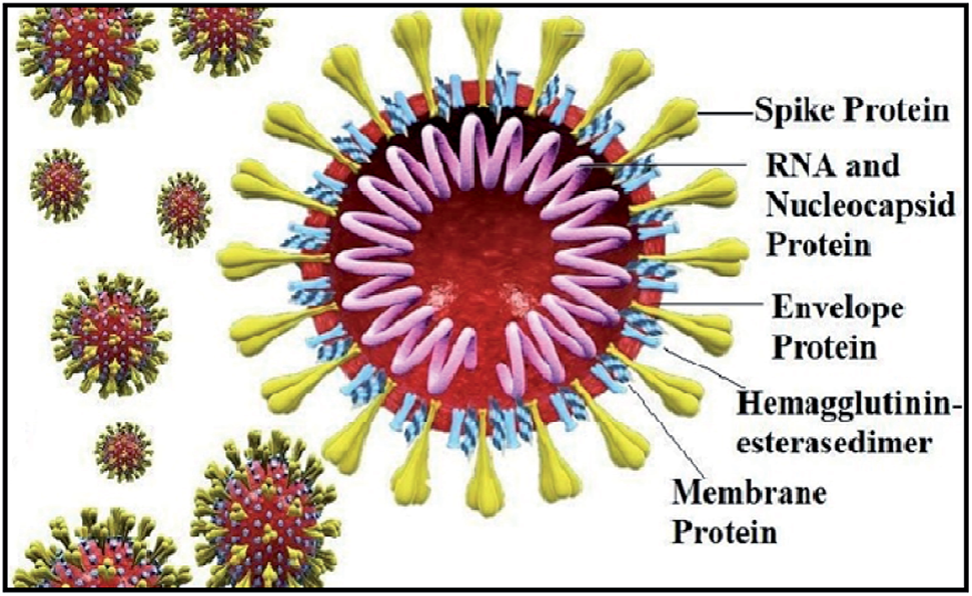
Structure of coronavirus showing its structural proteins.

The world has recently witnessed an outbreak of a potentially lethal coronavirus, nCovid-19, an urgent public health issue with an increase in morbidity and mortality. Coronaviruses belonging to the family Coronaviridae significantly threaten human health, and other species. Recent findings claim that the severe acute respiratory syndrome coronavirus 2 (SARS-CoV-2) is evolutionarily closely related to bat coronavirus and SARS-CoV with a homology of ≥95% and ≥70%, respectively.^1–3^ SARS-CoV-2 is a rapidly transmissible virus that tends to change its genetic material to survive in various environmental conditions. Structurally, this virus contains a nucleocapsid (N), where its genome is packed inside a helical capsid. A membrane protein (M) and small envelope protein play a major role in the virus assembly, and the spike glycoprotein or S protein plays a key role in host entry by harboring certain crucial amino acid residues of human angiotensin-converting enzyme 2 (*h*ACE2). The spike glycoprotein forms a large protrusion on the surface of the virus, exhibiting a crown-like appearance, and hence the name coronaviruses,^4–8^ as shown in Fig. 1.

Similar to SARS-CoV, SARS-CoV-2 also facilitates its entry into human angiotensin-converting enzyme 2 (ACE-2) through the receptor-binding domain (RBD) of the spike glycoprotein. Angiotensin-converting enzyme 2 (ACE-2), is an enzyme located on various parts of the body including alveolar epithelial cells of the lung, intestinal absorptive cells or enterocytes of small intestine, venous endothelial cells of the kidney, endothelial cells of the heart and renal tubular epithelial cells.^9^ Moreover, the binding affinity of SARS-CoV-2 on hACE2 is 10–20-fold higher than SARS-CoV-2002.^10^ Hence, targeting the entry of the SARS-CoV-2 spike glycoprotein RBD is considered a new therapeutic intervention.^11^ However, in the current pandemic situation, traditional drug discovery or vaccine development is a daunting and time-consuming task, which can be offset by computational-aided drug design.

**Fig. 2:**
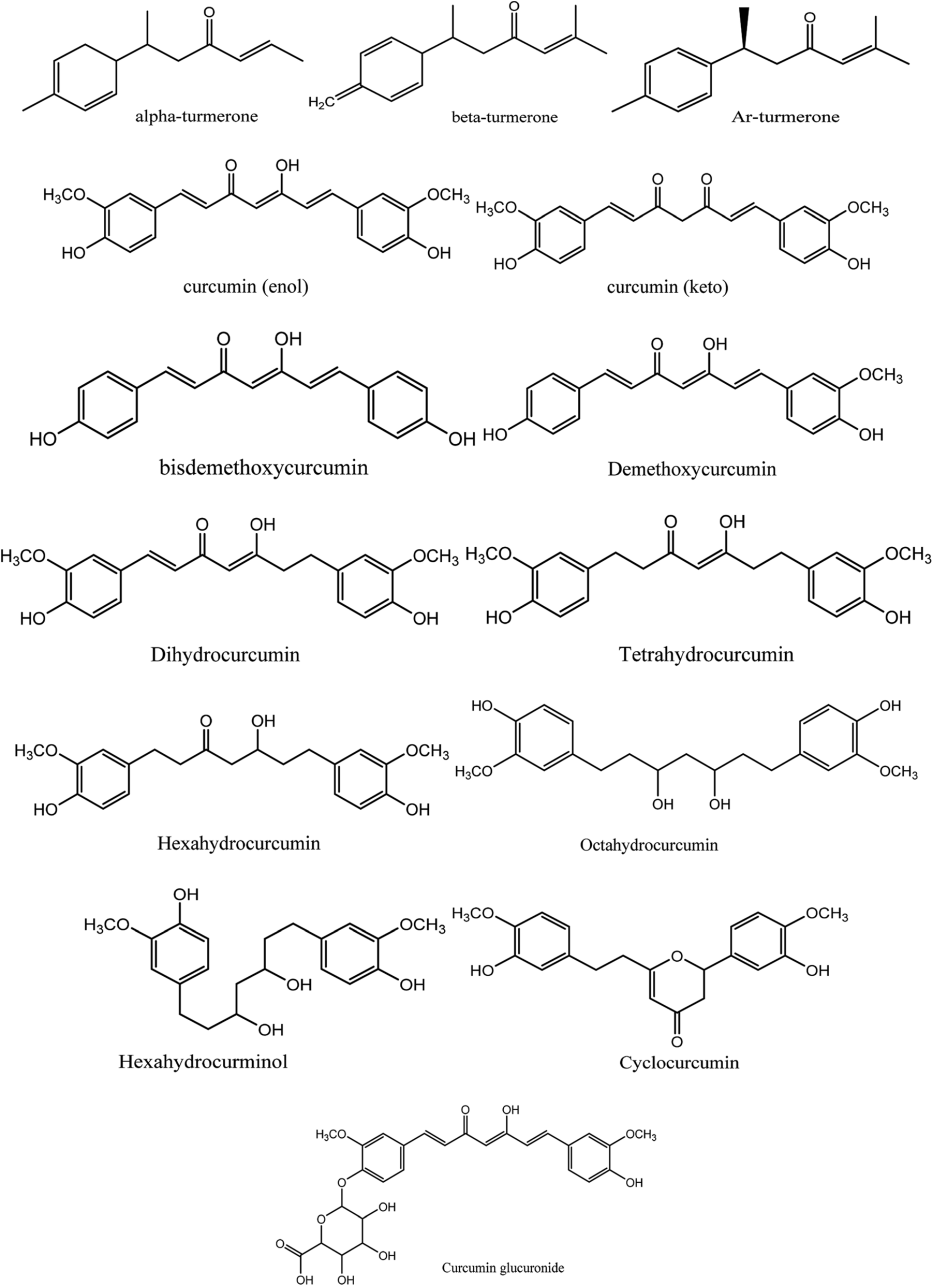
Chemical structures of 14 natural curcumin derivatives used in this study.

Moreover, instead of designing new lead compounds, drug repurposing or the use of natural products can be an alternative for the development of new antiviral compounds, which will enhance the speed of research in this area. In the current study, we focused on the natural plant *Curcuma longa*, commonly known as turmeric, a perennial herbaceous rhizomatous plant belonging to the ginger family Zingiberaceae, which is widely used in India.^12^ Curcuma longa chemical constitutes are widely used for treating various ailments and possess a wide variety of therapeutic properties including antiviral,^13^ analgesic,^14^ antimicrobial,^15^ antiproliferative,^16^ and anti-inflammatory^17^ activity (Fig. 2).

Among the various chemical compounds, curcumin has gained importance among researchers because its compounds have been exhibited activity against viruses such as the human immunodeficiency virus (HIV), dengue virus, herpes simplex virus (HSV), hepatitis virus, influenza A virus (IAV), and Ebola virus.^18–21^ Recently, reports on computational drug design demonstrated the therapeutic potential of curcumin as a dual inhibitory agent acting on S-protein and ACE-2.^22^ Thus, the main rationale of this study was to search for potential curcumin derivatives against these two drug targets, *i.e.* the spike glycoprotein RDB region and envelope protein. Besides, the pharmacokinetics, pharmacodynamics and drug-likeness of all the curcumin derivatives were studied. Finally, a molecular dynamics simulation study was performed to understand the stability of the protein–ligand complex with time, and a CHARMm interaction analysis and free energy binding using MM-PBSA demonstrated the stability of curcumin complexed with the spike glycoprotein.

## 2. Results and discussion

### 2.1 Homology modelling

**Fig. 3:**
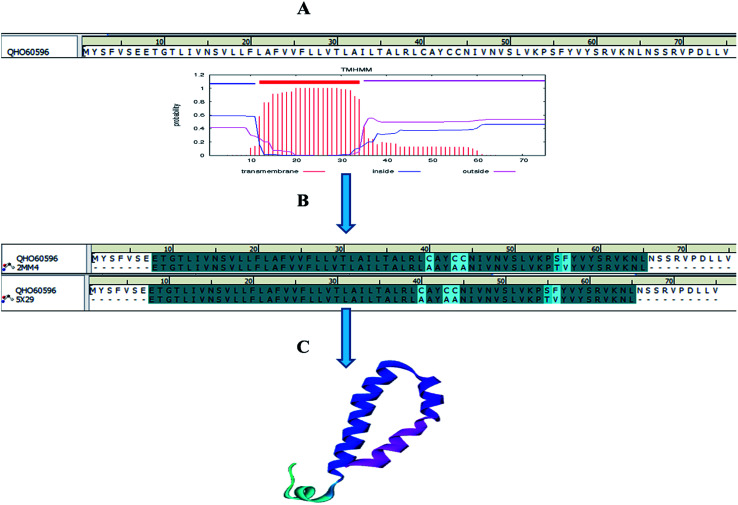
Envelope protein of SARS-CoV-2. (A) Transmembrane prediction, (B) query to template alignment, and (C) homology modelled structure.

Homology models are the backbone of structural biology, where a structure without NMR or X-ray crystallography data can be modelled using various machine learning algorithms. Moreover, it is a reliable and widely used technique for predicting unknown protein structures. The experimental protein structures of the novel coronavirus SARS-CoV-2 belonging to the genus Betacoronavirus are limited in structural databases. Therefore, the template modelling concept was implemented tsuences were scanned in the TMpred tool to identify the TM-alpha helices among the three query sequences, and only the envelope protein showed transmembrane helices (Fig. 3A).

**Fig. 4:**
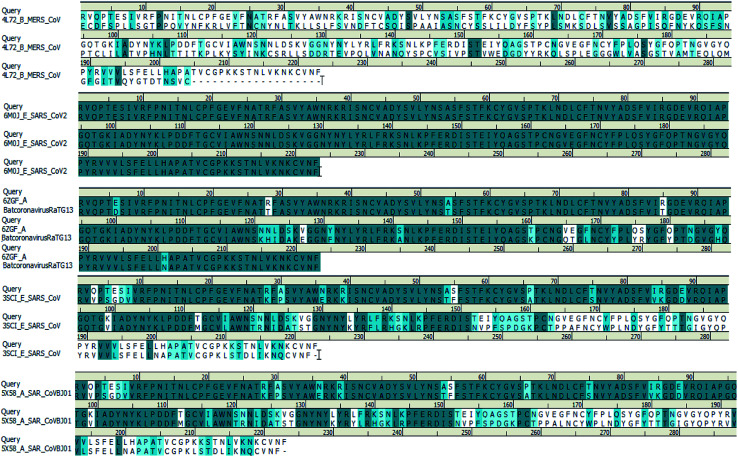
Spike glycoprotein_RBD query sequence of SARS-CoV-2 alignment with different template structures of MERS-CoV, batcoronavirusRaTG13, SARS-CoV and SARS-CoVBJ01.

We searched for templates for each query sequence using BLAST search against PDB databases. According to the BLAST search of the spike glycoprotein_RBD (333–526) region, it shows 100% template coverage with the RBD region of SARS-CoV-2 (PDB ID 6M0J_E) with a resolution of 2.45 Å. Also, the homology of bat coronavirus RaTG13 (PDB ID 6ZGF_A), SARS-CoV protein (PDB ID 3SCI_E), SARS-CoV BJ01 (PDB ID 5X58_A) and SARS-CoV-2 is about 90.13%, 73.54%, and 73.09% identity with query coverage of 100%, respectively. In contrast, the homology of the MERS S-protein (PDB ID 4L72_B) and SARS-CoV-2 S-protein RBD is about 24% identity and the query coverage rate is 44%. The BLAST search results of the query sequence to the PDB structure alignment is depicted in Fig. 4.

Subsequently, the BLAST search of the envelope protein (QHS34548) of SARS-CoV-2 showed the homology of SARS-CoV PDB ID's 2MM4_A and 5X29_A. SARS-CoV envelope protein 5X29_A shows maximum query coverage of 82% with 88.71% identity and 2MM4_A of SARS-CoV sequence alignment with a query of only about 77% with 91.38% identity. However, the aligning query sequence with individual templates of SARS-CoV using the MODELLER algorithm showed 83% sequence similarity and 81.5% sequence identity (Fig. 3B). Hence, template 5X29_A was used for homology modelling of the SARS-CoV-2 envelope protein. However, no template match was found with MERS-CoV. On the other hand, the membrane protein sequence for template structure identification showed no significant similarity results with the default scoring matrix of BLASTp. Consequently, other scoring matrices were introduced to check the structural similarity of the sequence, excluding an iterative process that showed no significant similarity to the query. Hence, the modelled envelope protein (Fig. 3C) and available X-ray crystallography structure 6M0J_E spike glycoprotein_RBD of SARS-CoV-2 were used for further studies.

### 2.2 Structure validation process

**Fig. 5:**
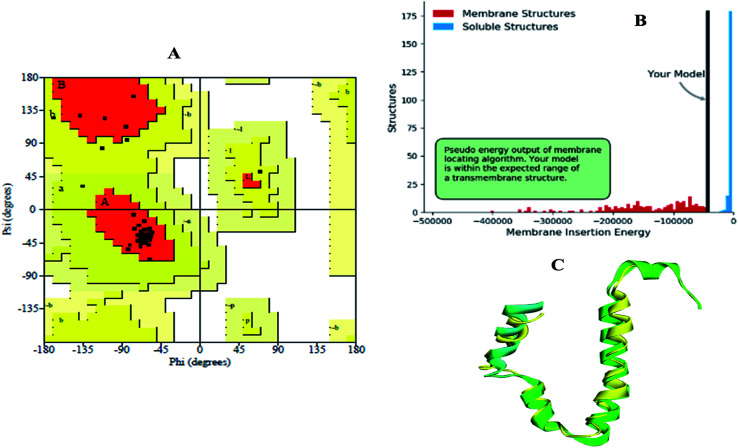
(A) Ramachandran plot, (B) transmembrane structure validation, and (C) superimposed model of modelled structure and template structure.

**Fig. 6:**
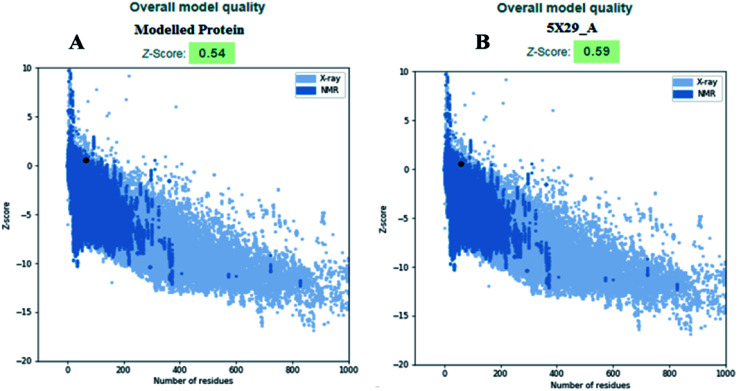
(A) Modelled envelope protein and (B) template structure used for modelling envelope protein.

The modelled envelope protein structure from the SARS-CoV structural parameters was studied for validation purposes. The best-modeled structure using MODELLER was initially chosen based on the lower discrete optimized protein energy (DOPE)^23^ of −4812.83 kcal mol^−1^, and the higher total PDF (probability density function) of 165.148 shows 92.98 ERRAT quality factor. The Procheck tool evaluates the residue-by-residue stereochemical quality, structure geometry and distribution of Phi and Psi angle amino acids in favoured, allowed and generously allowed region of a modelled protein.^24^ The Ramachandran plot (RP) showed 91.8% amino acids in the core region or most favoured regions and 8.2% residues were in the additional allowed regions with no outliers (Fig. 5A). Most of the amino acids in the favoured region signify that the modelled structure of the envelope protein is reliable, and can be equally compared with NMR structure quality. QMEANBrane is a unique structure quality assessing SWISS-MODEL tool explicitly used for modelled transmembrane protein investigation.^25^ The envelope protein probed in SWISS-MODEL was within the transmembrane insertion energy, and also satisfied statistical potentials in the naturally occurring oligomeric state (Fig. 5B). The template (5X29) and modelled structure superimposition are shown in Fig. 5C, where the overall structure root mean square deviation is <2.5 Å. The quality of the structure was further confirmed using the RAMPAGE tool. In addition, ProSA structure analysis^26^ was performed for the modelled protein and template structure, where the *Z*-scores of 0.54 and 0.59, respectively, located in the NMR structure region further confirm the modelled protein structure quality (Fig. 6).

### 2.3 Computational pharmacoinformatic study on curcumin derivatives

**Fig. 7:**
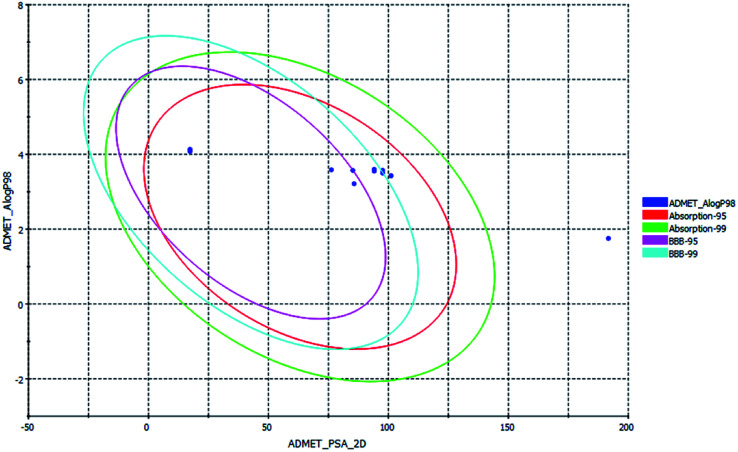
ADMET plot for curcuminoids, indicating their ideal properties.

ADMET, TOPKAT and drug-likeness assessment for lead molecules can provide insight into their quantitative structure–property relationship. Thus, the models used in the *in silico* pharmacokinetic study were obtained through a quantitative structure–activity relationship (QSAR). Furthermore, they reduce the research time, biological waste and cost. ADMET was initially used to a screen a large library of compounds. Descriptors such as the blood–brain barrier (BBB) penetration, hepatotoxicity and CYP2D6 enzyme are high priority models followed by Ames mutagenicity and carcinogenicity. All the curcuminoid compounds were tested using the ADMET model. The pharmacokinetic analysis results are shown in (Fig. 7). According to the ADMET plot Alog *p*_98 *vs.* PSA, it can be observed that compound 71315012 (curcumin glucuronide) is outside of the ellipses due to its low human intestinal absorption (HIA) and undefined BBB penetration, whereas some molecules such as 5318039 (hexahydrocurcumin), 11068834 (octahydrocurcumin) and hexahydrocurcumin are near to 99% confidence ellipses of the BBB, and the remaining compounds are inside 95% and 99% confidence ellipses of HIA. Also, the curcuminoids compounds exhibit good membrane permeability, except curcumin glucuronide, which has a polar surface area^27^ (PSA) of <140 Å^2^. Overall, the curcuminoids compounds are non-toxic to hepatic cells, non-inhibitors of a metabolic enzyme (CPYD26), and exhibit very high to medium penetration across the BBB except a few molecules (Table 1).

**Table 1:** *In silico* ADMET properties of curcuminoids

Compound name	Solubility	BBB	CPY2D6	Hepatotoxic	HIA	Alog *p*_98	PSA
Keto-curcumin	3	3	NI	NT	0	3.554	94.092
Dihydrocurcumin	3	3	NI	NT	0	3.577	94.092
Octahydrocurcumin	3	4	NI	NT	0	3.427	101.12
Tetrahydrocurcumin	3	3	NI	NT	0	3.6	94.092
Alpha-turmerone	2	0	NI	NT	0	4.079	17.3
Beta-turmerone	2	0	NI	NT	0	4.133	17.3
Bisdemethoxycurcumin	3	2	NI	NT	0	3.587	76.232
Hexahydrocurcumin	3	3	NI	NT	0	3.5	97.607
Demethoxycurcumin	3	2	NI	NT	0	3.57	85.162
Cyclocurcumin	3	2	NI	NT	0	3.213	85.722
Curcumin glucuronide	3	4	NI	NT	3	1.752	191.7
Enol-curcumin	3	4	NI	NT	0	3.573	97.607
Ar-turmerone	2	0	NI	NT	0	4.335	17.3
Hexahydroxycurcuminol	3	4	NI	NT	0	3.427	101.12

Similarly, the *in silico* pharmacodynamic models of the curcumin derivatives are free from carcinogens and mutagens. Quantitative-structure toxicity relationship (QSTR)-based toxicity parameters such as rat (TD50), rat_oral (LD50), rat_inhalation, fathead minnow (LC50) and *Daphnia* (EC50) are summarized in Table 2. With only one RO5 violation allowed, Alog *P* of ≤5, molecular weight of ≤500, hydrogen bond acceptors (HBA) of ≤10 and hydrogen bond donor (HBD) of ≤5 are also known as Lipinski's rule of 5 or Pfizer's rule of five, which is used to evaluate the drug-likeness property of lead molecules.^28^ Compounds that conform to these R05 rules may be pharmacologically and biologically active, in addition to Veber's rule of rotatable bonds (RB) of <10 and PSA of <140 Å^2^ (ref. 29) for orally bioactive compounds. Accordingly, all the compounds except curcumin glucuronide follow both Lipinski's and Veber's rule. Overall, 13 compounds possess oral bioavailability by obeying the drug-likeness rule tabulated in (Table 3).

**Table 2:** TOPKAT analysis of curcuminoids

Compound name	NTP carcinogen^a^	Ames mutagen^a^	Rat TD50^b^	Rat oral LD50^c^	Rat inhalation LC50^d^	Fathead minnow LC50^d^	*Daphnia* EC50^e^
Keto-curcumin	NC	NM	57.8227	2.81353	1200.80	0.000386	1.61229
Hexahydroxycurcuminol	NC	NM	1.53493	14.2048	1538.66	0.005549	0.481126
Dihydrocurcumin	NC	NM	63.4126	8.19582	1048.43	0.000318	1.05586
Octahydrocurcumin	NC	NM	1.53493	14.2048	1538.66	0.005549	0.481126
Tetrahydrocurcumin	NC	NM	25.0721	11.8342	1126.18	0.000875	0.600233
Alpha-turmerone	NC	NM	72.5357	1.09783	23297.40	0.000578	8.87751
Beta-turmerone	NC	NM	55.48	1.38263	26 058	0.000136	6.67372
Bisdemethoxycurcumin	NC	NM	168.944	1.00939	657.833	0.000988	3.7944
Hexahydrocurcumin	NC	NM	1.13932	8.65315	1963.47	0.004877	1.09273
Demethoxycurcumin	NC	NM	58.7615	2.39167	1645.26	0.000454	3.67163
Cyclocurcumin	NC	NM	24.1476	1.76033	3601.59	0.001092	1.03814
Curcumin glucuronide	NC	NM	1.42569	8.725	74.3232	0.004108	0.687055
Ar-turmerone	NC	NM	100.468	1.43641	16932.10	0.001595	26.6017
Enol-curcumin	NC	NM	53.0906	3.39621	927.783	0.000536	0.391523

a Non-Carcinogen (NC), Non-Mutagen (NM).

b TD50 (mg per kg body weight per day).

c LD50 (g per kg body weight), mg m^−3^ h^−1^.

d LC50 (g L^−1^).

e EC50 (mg L^−1^).

**Table 3:** Drug-likeness property of curcuminoids

Compound name	PubChem CID	Lipinski's rule of 5				Veber's rule	
		HBD	HBA	MW	Alog *P*	RB	PSA
Keto-curcumin	969516	6	2	368.38	3.554	8	93.06
Dihydrocurcumin	10429233	6	2	370.396	3.577	9	93.06
Octahydrocurcumin	11068834	6	4	376.443	3.427	10	99.38
Tetrahydrocurcumin	124072	6	2	372.412	3.6	10	93.06
Alpha-turmerone	14632996	1	0	218.335	4.079	4	17.07
Beta-turmerone	196216	1	0	218.335	4.133	4	17.07
Bisdemethoxycurcumin	5315472	4	2	308.328	3.587	6	74.6
Hexahydrocurcumin	5318039	6	3	374.428	3.5	10	96.22
Demethoxycurcumin	5469424	5	2	338.354	3.57	7	83.83
Cyclocurcumin	69879809	6	2	368.38	3.213	5	85.22
Curcumin glucuronide	71315012	12	5	544.504	1.752	11	189.28
Enol-curcumin	381330244	6	3	368.38	3.573	7	96.22
Ar-turmerone	558221	1	0	216.319	4.335	4	17.07
Hexahydroxycurcuminol	Sketched	6	4	376.443	3.427	10	99.38

### 2.4 Biological significance of receptor–ligand docking

Molecular docking is a key tool to understand the mode of binding of a compound to the active site of the target proteins. Two cell surface proteins are the main drug targets of novel SARS-CoV-2. A team of researchers working on the genus Alpha/Betacoronavirus has reported that each virus species uses different entry points to invade the human system, where the DPP4 and APN receptors are used by MERS-CoV^30^ and HCoV-229E, respectively.^31^ Similarly, the spike glycoprotein or surface glycoprotein or S-glycoprotein of SARS-CoV-2 and SARS-CoV uses the angiotensin-converting enzyme 2 (ACE2) receptor, a type-I transmembrane metallocarboxypeptidase, for cellular entry.^32,33^ It was recently reported that SARS-CoV-2 harbors the F486, N487, Y489, Q493, Q498, T500, N501, G502 and Y505 amino acid residues present in the receptor-binding motif (RBM) of the receptor-binding domain (RBD) of the S-glycoprotein to interact with the ACE2 receptor in humans.^1^

**Fig. 8:**
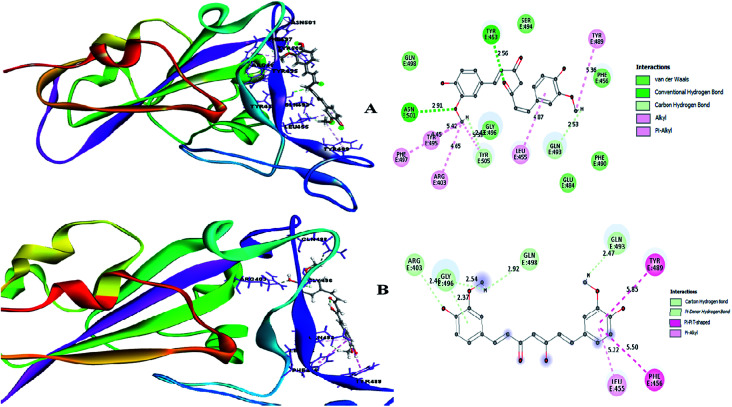
(A) Receptor–ligand interaction of S-glycoprotein (RBD) with the keto form of curcumin and (B) receptor–ligand interaction of S-glycoprotein (RBD) with the enol form of curcumin.

Therefore, the present study aimed to find the binding interaction of curcuminoid derivatives to key target residues of SARS-CoV-2 through docking studies using the CDOCKER algorithm, a grid-based CHARMm simulation tool. Among the 14 compounds docked to the site of RBM, only two forms of curcumin, namely, its keto and enol forms, interact to form strong hydrogen bond interaction with the key residues Q493, N501, Y505, Y489, and Q498 with the CDOCKER docking score of −20.753 kcal mol^−1^ and −16.8067 kcal mol^−1^ (Fig. 8A and B), respectively. Thus, the receptor–ligand interaction study indicated that both forms of the curcumin pharmacophore have the ability to interact with the spike glycoprotein, anchoring the residues of ACE2. Also, blocking these residues possibly does not facilitate its interaction with the human ACE2 receptor, and thereby viral infection can be controlled.

**Fig. 9:**
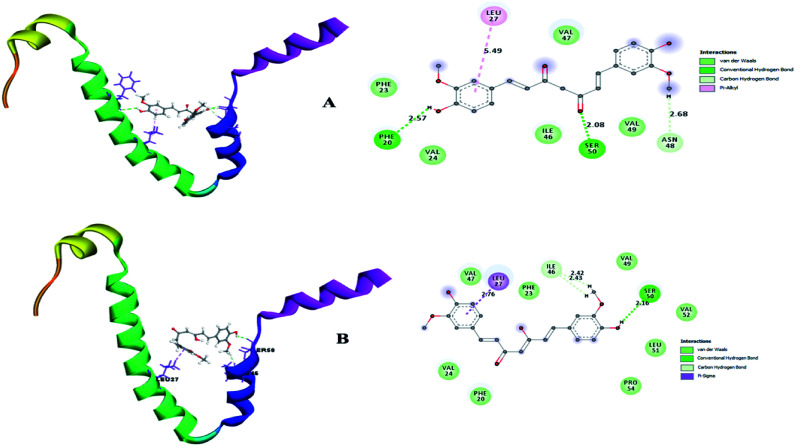
(A) Receptor–ligand interaction of S envelope protein with the keto form of curcumin and (B) receptor–ligand interaction of the envelope protein with the enol form of curcumin.

A small envelope protein (E-protein) is another drug target protein that plays three functional roles in SARS-CoV-2, including viral assembly,^34^ pathogenesis of the virus^35^ and release of virions.^36^ Targeting the E-protein functions aborts the viral assembly process, and consequently the formation of immature virions.^8^ On contrary, the exact function of the envelope protein is still enigmatic because various studies have shown that even without E-protein, the virus utilizes accessory proteins to form its core structure, but the virus efficiency is reduced by a hundred-to a thousand-fold during morphogenesis.^37^ During docking, it was observed that curcumin keto (−19.174 kcal mol^−1^) and enol (−14.115 kcal mol^−1^) interact with the TM alpha helix of the E-protein to form hydrogen and hydrophobic interactions (Fig. 9A and B), respectively. Thus, curcumin may be a candidate compound for treating SARS-CoV-2.

### 2.5 Receptor–ligand interaction pharmacophore generation for SAR analysis of curcumin derivatives

**Fig. 10:**
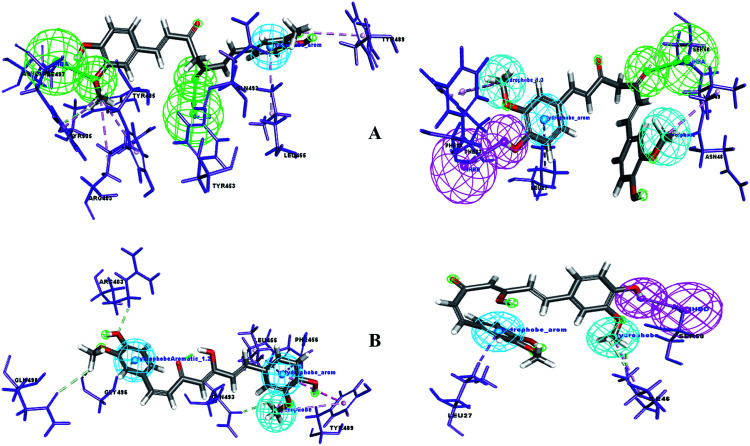
(A) Pharmacophore interaction of drug targets with the keto form of curcumin and (B) pharmacophore interaction of drug targets with the enol form of curcumin. Pharmacophore features green: Hydrogen Bond Acceptor (HBA), pink: Hydrogen Bond Donor (HBD), dark blue: hydrophobe aromatic, and cyan: hydrophobe.

A pharmacophore is a key tool to derive a structure–activity relationship (SAR) to distinguish the active functional groups of chemical compounds that are responsible for their biological activity. Curcumin is already known for its pleiotropism of pharmacological activity.^38^ Previously, the remarkable effect of dietary phenolic curcumin (1,3-dicarbonyl group) was reported for HIV drug targets, including integrase, Tat-mediated transactivation of the HIV-LTR and protease.^39^ In the current study, SAR was probed for two forms of curcumin, *i.e.* its keto form (1,3-dicarbonyl group) and enol form (1,3-keto–enol). To identify the probable functional moieties of curcuminoids, the receptor–ligand interaction complex was used to enumerate the pharmacophore. The pharmacophore interaction features generated for keto and enol curcumin are depicted in Fig. 10. It can clearly be observed that the keto form of curcumin shares a common HBA feature (=O) with two drug target proteins and no acceptor feature was mapped for enol curcumin, which contains =O and OH chemical groups (1,3-keto–enol moiety).

Uniquely, the keto form of curcumin containing the =O chemical group showed a tendency to bind with the active site amino acid in both drug targets. In contrast, in the enol form, the =O group is replaced with OH, which showed no binding interaction with the receptor sites. This reveals that the keto groups attached to the curcumin structure can be considered the important and crucial functional pharmacophore. Similarly, the enol form shares common pharmacophore hydrophobe aromatics and hydrophobes with the drug targets. Furthermore, to confirm this, the significance of substructure elimination of the –OCH_3_ moiety was studied. Accordingly, the removal of the OCH_3_ moiety in the curcumins showed no docking or interaction pharmacophore generation, which implies that together with the main core of curcumin, the OCH_3_ functional group is also required for binding interaction with the drug targets of SARS-CoV-2.

### 2.6 Binding energy and stability of the complex

Understanding the affinity of a compound to its target protein/receptors is the main objective of the structure-based drug design and drug discovery process. Specifically, the binding of a lead molecule to a receptor or signalling protein may alter its biological activity, and thus molecular structure recognition is considered a fundamental component in the virtual screening of drugs.

**Fig. 11:**
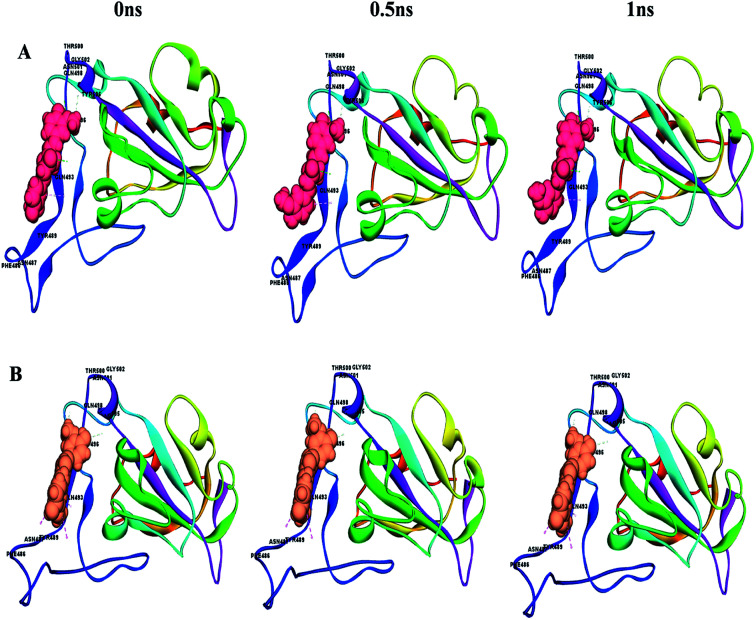
Conformational changes with time for (A) S-glycoprotein with the keto form of curcumin and (B) S-glycoprotein with the enol form of curcumin.

**Fig. 12:**
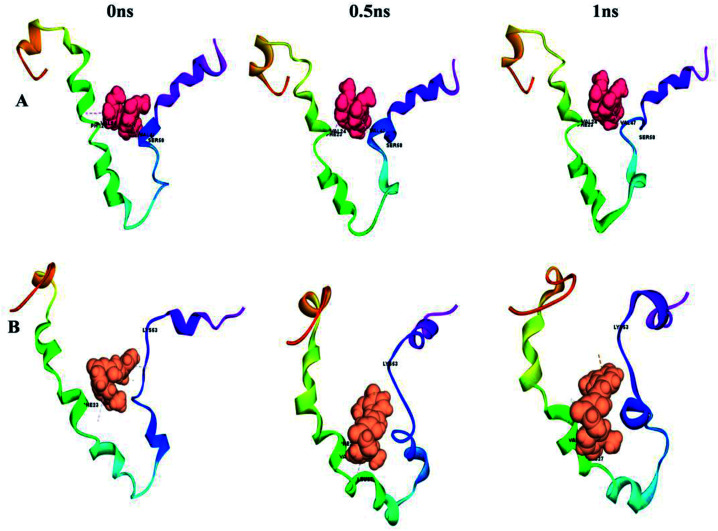
Conformational changes with time for (A) envelope protein with the keto form of curcumin and (B) envelope protein with the enol form of curcumin.

This can easily be achieved by means of calculating the binding energy of a docked receptor–ligand complex. The binding energy of the S-glycoprotein and envelope protein with the keto and enol forms of curcumin was computed using the equation (energybinding = energycomplex − (energyligand − energyreceptor)),^40^ where the negative energy of the binding complex shows the strength of the protein–ligand interaction (Table 4). A molecular dynamics study was carried out to study the stability of the receptor–ligand interaction. At the end of the study, binding site conformational changes were observed for the SARS-CoV-2 drug target proteins. The S-glycoprotein and envelope protein bound with the keto form of curcumin showed less secondary structural conformational changes compared to that with the enol form (Fig. 11 and 12), respectively. Further, significant flexibility and departure from the initial structure from molecular dynamics were estimated using the root mean square deviation (RMSD).

**Table 4:** Dynamics and binding energy parameters of the receptor–ligand complex

Energy parameters in (kcal mol^−1^)	S-glycoprotein		Envelope protein	
	Keto	Enol	Keto	Enol
Complex energy	−7378.69	−7350.24	1105.8	1129.009
Complex entropy	−29.9815	−29.9808	−28.1418	−28.1414
Binding energy	−56.57	−50.42	−51.71	−48.66
Potential energy	−10902.4	−10931.5	−2578.60	−2658.74
Total energy	−8630.45	−8679.34	−1741.24	−1725.18
Kinetic energy	2271.98	2252.18	837.431	833.556

**Fig. 13:**
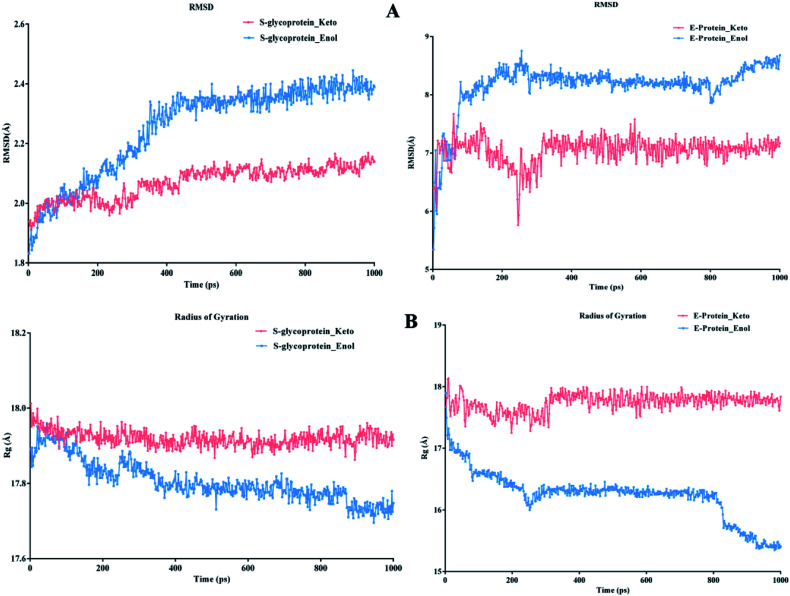
(A) RMSD graph for S-glycoprotein and envelope protein protein–ligand complex and (B) *R*_g_ graph for S-glycoprotein and envelope protein protein–ligand complex.

According to Fig. 13A, it can be observed that the RMSD deviation of the S-glycoprotein_keto deviations is within 2.1 Å, also the stability of the complex was maintained throughout the molecular dynamics study. In contrast, the S-glycoprotein_enol complex gradually changed to maintain its structural stability, but the deviation did not exceed 2.5 Å. Subsequently, the conformational fluctuation of the envelope protein with the keto and enol curcumin complexes showed a sharp peak at the beginning of the molecular dynamics. Conversely, after 0.2 ns, the conformational changes of both curcumin complexes were less up to the end of the molecular dynamics study. Another time-dependent analysis is the radius of gyration (*R*_g_), which is used to measure the compactness of proteins. Fig. 13B illustrates that the S-glycoprotein complexes with the keto and enol form showed negligible changes in protein structural folding, with *R*_g_ values in the range of 17.6 to 18. In contrast, the *R*_g_ value of the envelope protein with the enol form of curcumin changed over time, but at the end of the study, the structural compactness of the protein was retained, although the envelope protein complex with the keto form of curcumin showed less fluctuation and was stable throughout the molecular dynamics study. Thus, overall, the complex stability is relatively higher for the keto-bound complex than the enol complex.

### 2.7 Free and interaction energy parameter analysis for receptor–ligand complex

**Fig. 14:**
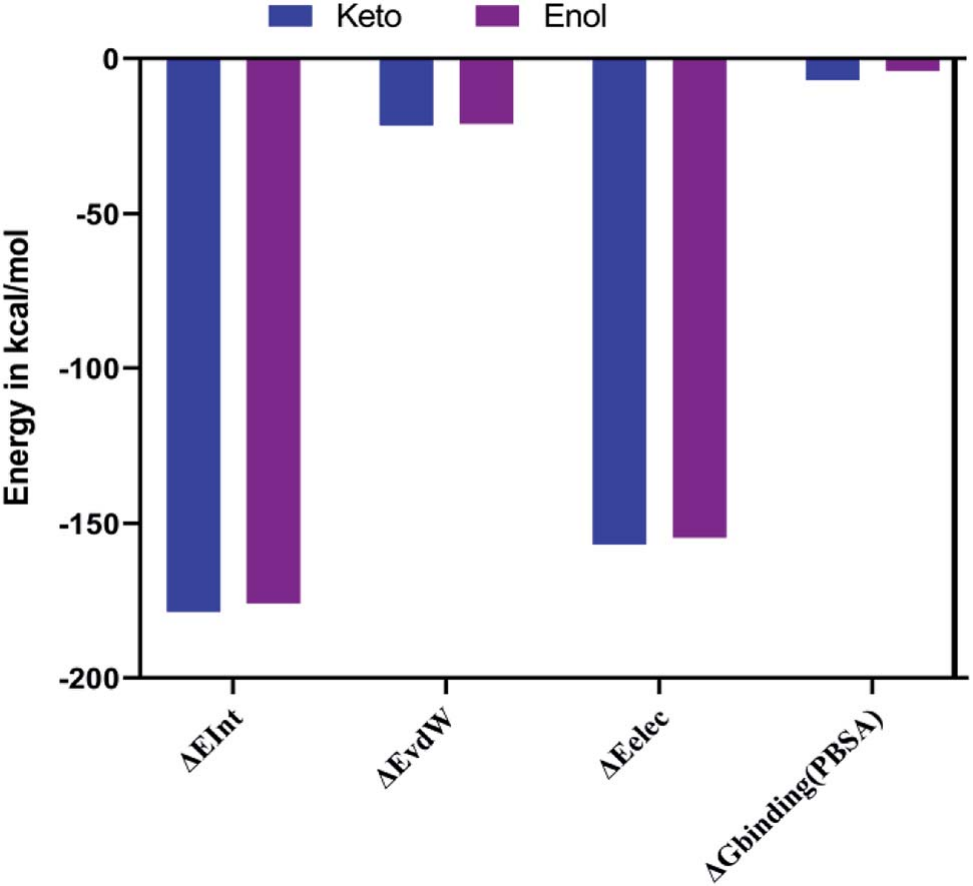
Graph showing the interaction (Δ*E*_Int_), van der Waals (Δ*E*_vdW_), electrostatic (Δ*E*_ele_), and free energy binding (Δ*G*_binding_) for both the keto-curcumin-S-protein RBD and enol-curcumin-S-protein RBD.

The combination approach of molecular mechanics energies with Poisson–Boltzmann surface area (MM/PBSA) is used to estimate the free energy of the binding of small ligands to biological macromolecules. The binding free energies for the two keto-curcumin-S-protein RBD and enol-curcumin-S-protein RBD complexes were estimated using the MM-PBSA method. The calculated binding free energy was −4.067 kcal mol^−1^ for enol-curcumin. Dongling *et al.*^41^ described that the favorable interaction of a complex can be measured in terms of electrostatic interaction. The analysis of the free energy components showed the that the binding free energy of −7.006 kcal mol^−1^ and more favorable electrostatic interaction (Fig. 14) for the keto-curcumin-S-protein RBD complex are the main reason for the higher affinity of keto-curcumin compared to enol-curcumin. On the other hand, the van der Waals interaction is more favorable for both the keto-curcumin and enol-curcumin conformations by −21.671 kcal mol^−1^ and −21.168 kcal mol^−1^, respectively. Finally, the interaction energy of the complex was calculated using the CHARMm energy, which exhibited a 0.98-fold change difference for keto to enol curcumin. Comparatively, all the energy values are more favorable for both forms of curcumin with slight fold changes in energy values.

## 3. Conclusion

SARS-CoV-2 has resulted in a devastating pandemic with global concern; however, present therapies in virology fail to prevent its effects. Currently, there is exigency in identifying novel leads with anti-viral properties to impede viral pathogenesis in the host system. Thus, two important curcuminoids of turmeric, *i.e.*, its curcumin keto and enol forms, were demonstrated to be complementary to bind with the S-glycoprotein and envelope protein of SARS-CoV-2. However, the keto form of curcumin is more favourable for both these drug targets considering its docking score, binding energy and molecular dynamics simulation. Thus, this study indicates that surface proteins are key drug target proteins of SARS-CoV-2, and probably curcumin blocks essential biologically active drug target residues, thereby attenuating the viral infection. Hence, this computational biology approach identifies curcumin as a drug candidate for further investigation in treating SARS-CoV-2. However, this was an initial study to identify the active pharmacophore of the compound and its binding site structural complementary for two drug targets. Thus, in the future, we aim to perform large-scale molecular dynamics, and *in vitro* and *in vivo* experiments to confirm the efficacy of curcumin against SARS-CoV-2.

## 4. Materials and methods

### 4.1 Homology modelling

The coronavirus protein sequences reported in the Indian State of Kerala such as the envelope protein (QHS34548), membrane protein (QIA98586) and spike glycoprotein_RBD region (QIA98583) structures not reported in the PDB databases were determined using the template-based modelling technique. All the sequences were downloaded from the National Genomics Data Center (https://bigd.big.ac.cn/ncov/). Two approaches of a basic local alignment search tool for protein (BLAST_p) were performed, where one was a direct hit on a server (https://blast.ncbi.nlm.nih.gov/Blast.cgi) and the other technique was through the BLAST_NCBI search protocol in Biovia DS2019. The template structures were selected by considering maximum identity and query coverage with less positive and *E*-values, where template hits with SARS-CoV/MERS-CoV species were given preference. Query sequences were aligned with the template structure followed by build homology modelling using MODELLER 9.17v9.^42^

### 4.2 Modelled structure validation

The homology model protein was subjected to quality assessment in various structural validation servers and tools. In Discovery Studio, the best model structure was selected based on three parameters, including superimposing the best model structures with the PDB template structure to calculate the root mean square deviation using the align and superimpose protein protocol. Statistically, a lower DOPE score and high PDF total energy represent the best quality of model structures with stable conformations. Besides, external web servers such as ERRAT (https://servicesn.mbi.ucla.edu/ERRAT/), Procheck (https://servicesn.mbi.ucla.edu/PROCHECK/), and Rampage (http://mordred.bioc.cam.ac.uk/∼rapper/rampage.php) were used to assess the structure quality. Some structures that showed more outliers in the Ramachandran plot were further optimized by the energy minimization technique provided with an RMS gradient of 0.1 kcal mol^−1^ Å^−2^ to prevent bad steric contact of atoms.^43^ Besides, a deficiency in side chain and loop amino acids was processed using side and loop refinement by CHARMm simulation and force field.^44^ The best-validated structures were used for further docking analysis.

### 4.3 Chemical structure preparation

The natural form of 14 curcumin derivative compounds were retrieved from the PubChem compound and substance database (https://pubchem.ncbi.nlm.nih.gov/). All compounds were prepared using the ligand protocol to generate various 3D conformations, isomers, and tautomers of the compounds to remove duplicity and to fix bad valances.

### 4.4 ADMET, drug-likeness and toxicity predictions

The curcumin derivative was submitted to the ADMET and TOPKAT tools of small molecule protocol for the *in silico* pharmacokinetics and pharmacodynamic studies. The p*K*_a_ study included parameters such as human intestinal absorption (HIA), aqueous solubility, blood–brain-barrier penetration (BBB), cytochrome CYP2D6 inhibition, plasma protein binding (PPB) and hepatotoxicity. In the pharmacodynamic study, we included animal models such as NTP rodent carcinogenicity, rat_oral LD50, rat_TD50, fathead minnow LC50, *Daphnia* EC50, rat inhalational LC50 and Ames mutagenicity. All these models were developed and validated based on a quantitative-structure toxicity relationship (QSTR).

### 4.5 Molecular docking and binding energy

**Fig. 15:**
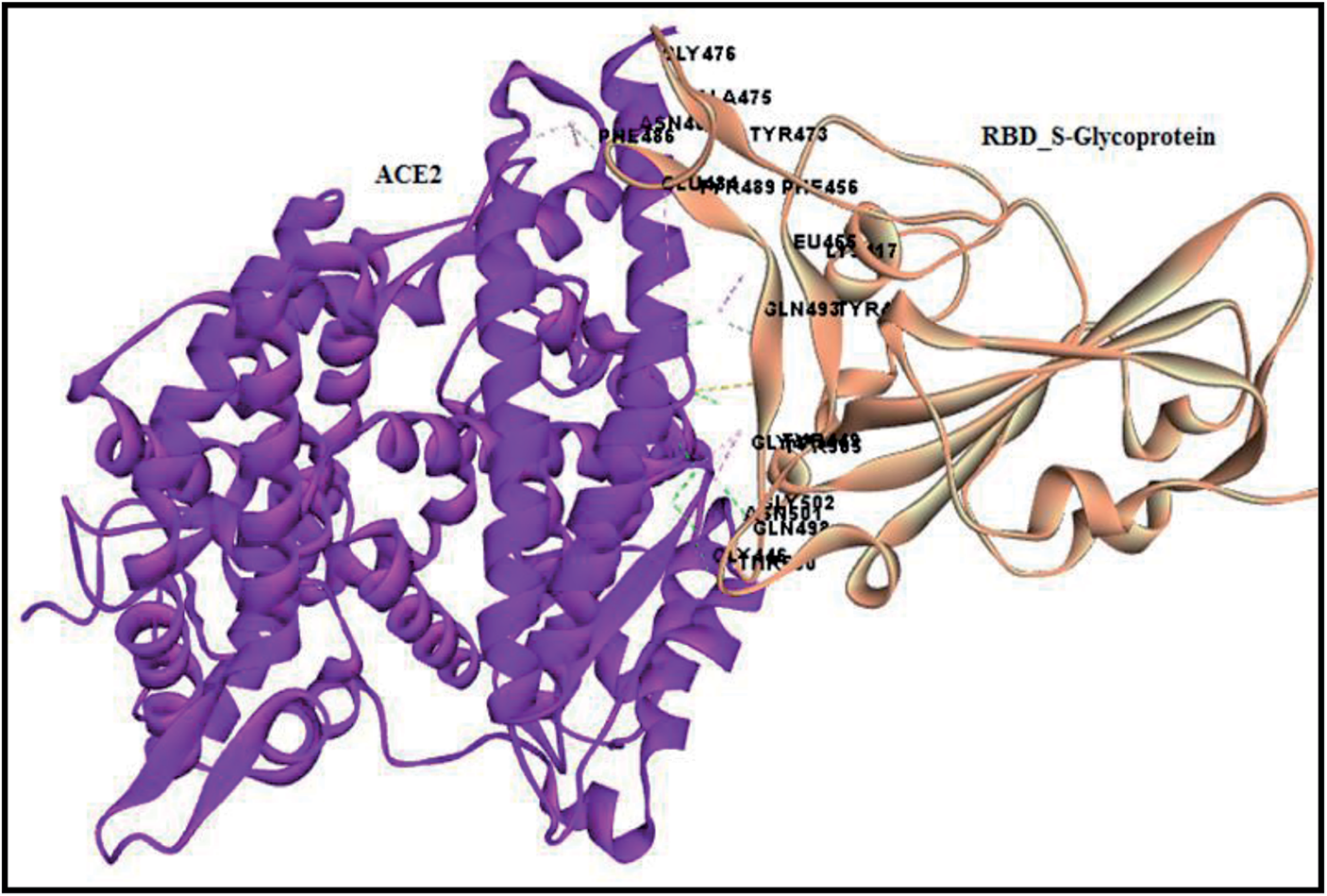
Analyzed interface residues of ACE2 RBD region of S-glycoprotein in SARS-COV-2.

Molecular docking is a lock and key process to identify compounds with complementary structures to drug target proteins. The CDOCKER algorithm is a grid-based and molecular dynamic simulation-implemented docking protocol, which was utilized for docking compounds to the binding site of the protein. The spike glycoprotein and envelope proteins are two novel drug targets for the dreadful covid-19 virus, which are the binding sites identified using a receptor-based cavity tool in Discovery Studio 2019. However, the spike glycoprotein (S-glycoprotein) RBD region binding to ACE2 receptor interactions were analyzed using the analyze protein interface tool in macromolecules to spot hydrogen, hydrophobic and other interactions, which was considered as the binding site for compound docking (Fig. 15). Both proteins were placed in an equal grid spacing in right angles in the 3D direction of the input site sphere coordinates with the radius of −38.36*X*, 31.32*Y*, 3.33*Z*, 20.1 Å and 37.37*X*, 33.46*Y*, 17.81*Z*, 26.873 Å for the RBD region of the S-glycoprotein and envelope protein, respectively. The docked complexes were further analyzed and validated *via* the negative CDOCKER energy and receptor–ligand interactions. Further, to understand and quantify the strength of the interactions between a ligand and protein, the binding energies between all the docked poses of the ligand with receptor were studied by utilizing the binding energy protocol with CHARMm. This is a crucial process in drug discovery and lead optimization. The best pose with the lowest binding energy was considered for the molecular dynamics simulation.

### 4.6 Molecular dynamics and simulation

The top compounds with the best pose in receptor–ligand interaction for the two different drug target proteins after docking validation were selected for molecular dynamics simulation. The whole system was subjected to CHARMm force field to satisfy bonded and non-bonded interactions, followed by standard dynamics cascade in a five-step simulation protocol. Initially, two 500 steps of minimization were performed using the steepest descent and conjugate gradient.^45^ The minimized complexes were gradually driven from 50 K to a final target temperature of 300 K, followed by equilibration simulations. Finally, production was performed for 1000 ps for four complexes. The leapfrog dynamics integrator and shake constraint were introduced throughout the molecular dynamics simulation to study the bonded and non-bonded interaction. The molecular dynamics trajectory was determined for complex stability and time-dependent analysis (RMSD and radius of gyration (*R*_g_)) using the Biovia Discovery Studio 2019 analyze trajectory protocol (Dassault Systèmes).^46^

### 4.7 CHARMm interaction energy and molecular mechanics-Poisson–Boltzmann surface area (MM-PBSA) analysis

This study was focused on the impact of curcumoinds on the stability of the S-protein. Thus, the complex structure of keto-curcumin-S-protein RBD and enol-curcumin-S-protein RBD was used as a starting point for calculating the binding free energies. The 1000 ps molecular dynamics simulation was carried out using CHARMm. In CHARMm, molecular dynamics simulations are performed using a classical mechanics approach, in which Newton's equations of motion are integrated for all atoms in the system.^47^ Thus, for each MD-simulated complex, we calculated the free binding energy (Δ*G*_binding_) values for the 500 conformations of the MD trajectory. During the simulation, one conformation or snapshot was saved every 2 ps up to 1000 ps, and the final Δ*G*_binding_ is the average of 500 conformations of the receptor–ligand complex. In the MM-PBSA method, the free energy of the protein–ligand binding (Δ*G*_binding_) is obtained from the difference between the free energies of the protein–ligand complex (*G*_complex_) and the unbound receptor/protein (*G*_protein_) and ligand (*G*_ligand_) as follows:Δ*G*_binding_ = Δ*G*_complex_ − [Δ*G*_protein_ + Δ*G*_ligand_]

The output conformations of the molecular dynamics simulation were further sampled to study the interaction energy between sets of atoms across all conformations using CHARMm. The interface interacting residues of the receptor and ligand were selected as two atom sets together with a cut-off distance of 12–10 Å to calculate the non-bonded interactions as follows: Δ*E*_Int_ = Δ*E*_vdW_ + Δ*E*_ele_where, Δ*E*_Int_=interaction energy of ligand–protein, Δ*E*_vdW_ = van der Waals energy of ligand–protein, and Δ*E*_elec_ = electrostatic energy of ligand–protein.

## Funding

This research project has not received any funding from any agencies.

## Conflicts of interest

The authors confirm that this article content has no conflicts of interest.

## Supplementary Material
